# Applying the en-bloc technique in corpus callosum glioblastoma surgery contributes to maximal resection and better prognosis: a retrospective study

**DOI:** 10.1186/s12893-023-02264-4

**Published:** 2024-01-02

**Authors:** Tianshi Que, Xi Yuan, Jian-er Tan, Haojie Zheng, Guozhong Yi, Zhiyong Li, Xiaoyan Wang, Junlu Liu, Haiyan Xu, Yajuan Wang, Xi-an Zhang, Guanglong Huang, Songtao Qi

**Affiliations:** 1 Department of Neurosurgery, Nanfang Hospital, Southern Medical University, Guangzhou, 510515 Guangdong People’s Republic of China; 2 The Laboratory for Precision Neurosurgery, Nanfang Hospital, Southern Medical University, Guangzhou, 510515 Guangdong People’s Republic of China; 3 Nanfang Glioma Center, Nanfang Hospital, Southern Medical University, Guangzhou, 510515 Guangdong People’s Republic of China; 4 Nanfang PET Center, Nanfang Hospital, Southern Medical University, Guangzhou, 510515 Guangdong People’s Republic of China

**Keywords:** Corpus callosum glioblastoma, En-bloc technique, Supramaximal resection, Neurological function, Survival

## Abstract

**Background:**

Corpus callosum glioblastoma (ccGBM) is a specific type of GBM and has worse outcomes than other non-ccGBMs. We sought to identify whether en-bloc resection of ccGBMs based on T2-FLAIR imaging contributes to clinical outcomes and can achieve a satisfactory balance between maximal resection and preservation of neurological function.

**Methods:**

A total of 106 adult ccGBM patients (including astrocytoma, WHO grade 4, IDH mutation, and glioblastoma) were obtained from the Department of Neurosurgery in Nanfang Hospital between January 2008 and December 2018. The clinical data, including gender, age, symptoms, location of tumor, involvement of eloquent areas, extent of resection (EOR), pre- and postoperative Karnofsky Performance Status (KPS) scales, and National Institute of Health stroke scale (NIHSS) scores were collected. Propensity score matching (PSM) analysis was applied to control the confounders for analyzing the relationship between the en-bloc technique and EOR, and the change in the postoperative KPS scales and NIHSS scores.

**Results:**

Applying the en-bloc technique did not negatively affect the postoperative KPS scales compared to no-en-bloc resection (*P* = 0.851 for PSM analysis) but had a positive effect on preserving or improving the postoperative NIHSS scores (*P* = 0.004 for PSM analysis). A positive correlation between EOR and the en-bloc technique was identified (*r* = 0.483, *P* < 0.001; *r* = 0.720, *P* < 0.001 for PSM analysis), indicating that applying the en-bloc technique could contribute to enlarged maximal resection. Further survival analysis confirmed that applying the en-bloc technique and achieving supramaximal resection could significantly prolong OS and PFS, and multivariate analysis suggested that tumor location, pathology, EOR and the en-bloc technique could be regarded as independent prognostic indicators for OS in patients with ccGBMs, and pathology, EOR and the en-bloc technique were independently correlated with patient’s PFS. Interestingly, the en-bloc technique also provided a marked reduction in the risk of tumor recurrence compared with the no-en-bloc technique in tumors undergoing TR, indicating that the essential role of the en-bloc technique in ccGBM surgery (HR: 0.712; 95% CI: 0.535–0.947; *P* = 0.02).

**Conclusions:**

The en-bloc technique could contribute to achieving an enlarged maximal resection and could significantly prolong overall survival and progression-free survival in patients with ccGBMs.

**Supplementary Information:**

The online version contains supplementary material available at 10.1186/s12893-023-02264-4.

## Background

Corpus callosum glioblastomas (ccGBMs), which are defined as glioblastomas invading and/or crossing the corpus callosum to the contralateral hemisphere, have worse outcomes than other non-ccGBMs [1]. The primary reasons are the essential role of the corpus callosum and the deep location of tumors making it hard to achieve radical resection. In this condition, the median overall survival for ccGBMs with surgical resection is only 7.0–15.0 months [1–4]. Therefore, a satisfactory resection with a low incidence of complications is worth to constantly exploring for neurosurgeons.

Many studies have reported that a large proportion of patients undergo biopsies with or without chemoradiotherapy [5, 6], and the median survival is only several months. The extent of resection (EOR) is positively related to survival time and clinical outcomes in glioma patients [7–10], including ccGBMs [5]. Recently, it was reported that maximal resection of T2-FLAIR abnormal signals contributed to benefiting patients’ survival and reducing tumor burden and recurrence with no additional risk of neurologic deficits in gliomas [11–13]. However, the distinctive location of ccGBMs limits resection based on T2-FLAIR imaging. Thus, in previous studies on ccGBMs, the evaluation of tumor resection was mainly based on contrast-enhanced imaging.

Corpus callosum glioblastomas generally have two parts: the lobar part and the corpus callosum part. We consider that the resection of the lobar part could be based on T2-FLAIR imaging, as we did in frontal gliomas [14]. In this retrospective study, we evaluated a series of ccGBMs from our institution over 10 years. General clinical data, EOR, postoperative complications and survival data were collected and analyzed. The primary goals of this study are to identify that resection of the lobar part of ccGBMs based on T2-FLAIR imaging contributes to clinical outcomes with an acceptable incidence of complications. Moreover, we also attempted to display our surgical strategy and aggressive surgical resection in ccGBMs, which did not affect the satisfactory balance between the maximal resection and the preservation of neurological function.

## Methods

### Patient selection and clinical data collection

A total of 106 adult ccGBM patients (including astrocytoma, WHO grade 4, IDH mutation and glioblastoma) were obtained from the Department of Neurosurgery in Nanfang Hospital between January 2008 and December 2018. Patients with > 80 years old and with multifocal tumor or gliomatosis cerebri (≥ 3 lobes of the brain affected) were excluded preoperatively. All patients in this study had not previously received any tumoral treatment. Written consent for data collection was obtained from every patient. Patients with any enhanced lesions left as seen on CE images after surgery and those who refused subsequent adjuvant therapy were also excluded. Three surgeons (A, B and C) performed the surgery, and the series could be divided to two subgroups: the en-bloc resection and piecemeal resection subgroups. Clinical data, including gender, age, symptoms, location of tumor, involvement of eloquent areas, EOR, pre- and post-operative Karnofsky Performance Status (KPS) scores, pre- and postoperative National Institute of Health stroke scale (NIHSS) scores, were collected (Supplementary Table 1). For the investigative use of these clinical materials, prior consent from patients and approval from the Ethics Committees of Nanfang Hospital were obtained.

### Radiological profiles and assessment of extent of resection

Preoperative magnetic resonance imaging (MRI) contained T1WI, T2WI, fluid attenuated inversion recovery (FLAIR), diffusion-weighted imaging (DWI), T1-contrast enhanced (CE), diffusion tensor imaging and magnetic resonance spectroscopy sequences. Postoperative MRI, including T1WI, T2WI, FLAIR, DWI, T1-CE and diffusion tensor imaging, was performed within 72 hours after surgical resection in all cases. Tumor or abnormal signal volumes were measured using pre- and postoperative T1-CE and FLAIR. The DWI sequence was used to confirm whether the postoperative FLAIR abnormal signal was residual tumor or surgically induced edema or infarction [15]. The extent of resection included supramaximal resection (SMR), total resection (TR) and subtotal resection (STR). The criterion of SMR was the follows: all FLAIR abnormalities were removed in the relative lobe and all CE abnormalities were resected in the corpus callosum part. TR was defined as all CE abnormalities were removed both in the lobar and the corpus callosum parts but had residual signal abnormalities on FLAIR images in the lobar part. STR was considered as any enhanced lesions left on CE images, but cases with STR were excluded from this study. Two neuroradiologists, blinded to the patients’ outcomes, separately reviewed the MRI scans to identify EOR in each case.

### Surgical highlights

**Fig. 1:**
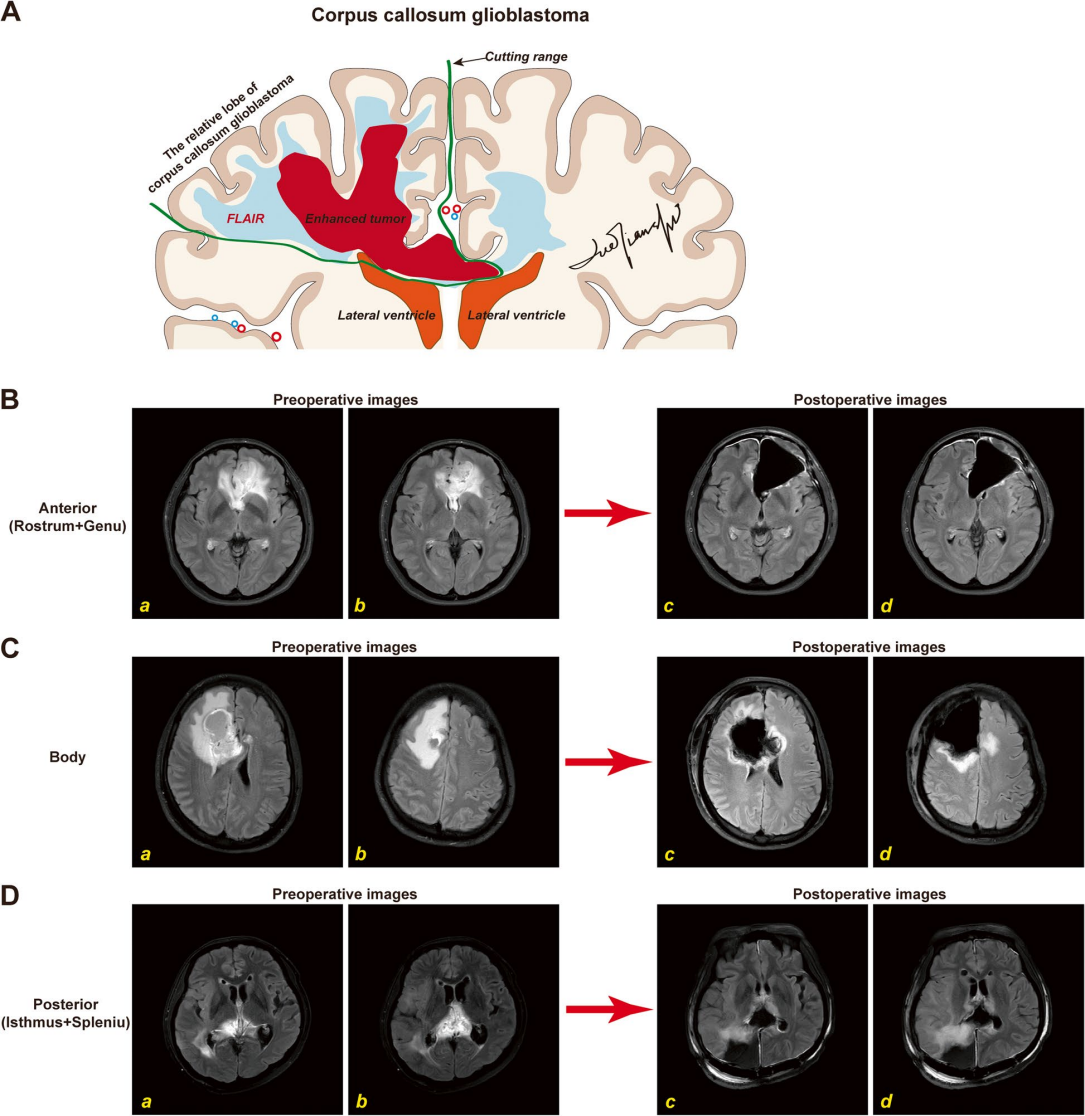
The surgical strategy of corpus callosum glioblastomas in Nanfang Neurosurgery. **A** Diagrammatical representation of the strategy and the techniques in resection of corpus callosum glioblastomas (ccGBMs) in a sagittal view. The tumor is removed by the en-bloc technique. The lobar part of ccGBM was dissected according to FLAIR images, and we used membranous structures including the pia mater and ependyma, as the surgical margins. The cortical cutting edge was the anatomically adjacent sulcus next to the gyri, which presented an abnormal signal on FLAIR images. In addition, we highlight the removal of the ventricle wall in ccGBMs. *The red mass represents the enhanced tumor, and the light blue region around the enhanced tumor represents the FLAIR region*. *The green curve represents the excisional range. The small red circles represent the arteries, and the small blue circles represent the veins. The schematic diagram was drawn by Tianshi Que.* **B-C** Typical cases. A middle-aged female with IDH1 wild-type ccGBM, in which the tumor was located in the anterior of the corpus callosum and was mainly related to the left frontal lobe, underwent supramaximal resection with wide ventricle wall resection by the en-bloc technique (**B**). A middle-aged male with an astrocytoma, WHO grade 4, IDH mutation, which is in the body of the corpus callosum and was mainly related to the right frontal lobe (**C**). A young female with IDH1 wild-type ccGBM in the posterior corpus callosum underwent supramaximal resection with removal of the relatively right occipital lobe (**D**)

A schematic diagram of the surgical strategy is shown in Fig. 1A, and typical cases are shown in Fig. 1B-D. The principle of surgery was maximal resection of tumors, on the condition of avoiding severe complications, and if not, loss of function did not impede subsequent adjuvant therapy after resection. The corpus callosum part of the tumor was removed along a gliotic pseudoplane between the tumor and brain tissue. The lobar part of the tumor was removed by lobectomy or according to the following principles: the surgical margin was identified by anatomical and functional boundaries, and the cortical cutting edge was the anatomically adjacent sulcus next to the gyri, which presented an abnormal signal on FLAIR images [14].

For tumors involving the rostrum and the genu of the corpus callosum, the key point of the surgery was preserving the anterior cerebral artery system based on utilizing the arachnoid membrane of the interhemispheric cistern to guarantee fewer complications. For the tumors that involved the isthmus and splenium of the corpus callosum, the critical points of the parietooccipital part surgery were preservation of the deep cerebral venous system and application of the ependyma to avoid damage to the thalamus and the diencephalon, as we described [16]. For tumors involving the body of the corpus callosum, surgeons should focus on preserving the artery supplying the cortex of the central gyrus, and the central gyrus itself.

### Postoperative management

The postoperative management followed the National Comprehensive Cancer Network Clinical Practice Guidelines. Regardless of the molecular profile, patients with glioblastomas underwent standard radiotherapy plus concurrent temozolomide and adjuvant temozolomide. MRI was performed every 3 months to monitor tumor progression.

### Outcomes

Postoperative neurological situation was evaluated by the NIHSS score. The neurological deficit was defined as a loss of at least a 1-point NIHSS score. The assessment was performed before surgery and at 3 months after surgery. Overall survival (OS) was recorded as the time from the first surgery to the time of death for those patients who died of any cause or to the time of last follow-up or end of study (June 30, 2022) for surviving patients. Progression-free survival (PFS) was considered as the time from first surgery to recurrence, or as the time of last follow-up or end of study (June 30, 2022) for recurrent-free patients.

### Statistical analysis

Statistical results were analyzed using IBM SPSS v26.0 and GraphPad Prism v9.0 software. The statistical methods were similar to those previously described [14]. Descriptive data were expressed as *n* (%), mean ± standard deviation. Statistical significance was calculated using the two-tailed *t* test for two groups and one-way ANOVA for multiple groups. The chi-square test or Fischer’s exact test was used to identify differences between categorical variables. The results were adjusted by Bonferroni correction and/or the Dunnett method to avoid the risk of Type I errors, and the new threshold for statistical significance after correcting for multiple comparisons was indicated in the manuscript. To analyze the relationship between the en-bloc technique and EOR, and the change in the postoperative KPS scales and NIHSS scores, propensity score matching (PSM) analysis was applied to control for confounders. For propensity score calculation, logistic regression was used with the en-bloc technique as a dependent variable as a function of gender, age (continuous), tumor volume, tumor location, pathology, preoperative KPS and preoperative NIHSS (continuous), and with a 0.2 caliper width. The match ratio was 1:1. Survival analysis was performed using the Kaplan-Meier method based on the datasets after propensity score calculation. Univariate and multivariate Cox proportional hazards methods were used to analyze the relationship between variables and OS or PFS. The results of Cox regression were reported as hazard ratios with 95% confidence intervals (CI). All differences were considered statistically significant for *P* < 0.05, unless otherwise indicated.

## Results

### Patient demographics, clinical presentation and pathological findings

A total of 106 primary ccGBM patients underwent surgery between January 2010 and December 2020. The subjects comprised 59 males and 41 females, with ages ranging from 22 to 71 years (mean, 49.13 years). Based on our previous study, all patients were divided into three age groups (18–47 years old, 48–63 years old and 64–75 years old), which established the age group classification for risk stratification in glioma patients [17]. Forty patients (37.7%) had ages ranging from 18 to 47 years and 51 patients (48.1%) were in the 48–63 years group, whereas 15 (14.2%) patients belonged to the 63–75 group. The mean tumor volume was 56.0 ± 17.3 cm^3^, ranging from 28.4 to 105.3 cm^3^. Based on the anatomical features of the corpus callosum [18], all ccGBM cases could be divided into the following three subgroups: anterior (rostrum and genu), posterior (isthmus and splenium) and body (the body of the corpus callosum) groups. Fifty-seven (53.8%), 24 (22.6%) and 25 (23.6%) cases belonged to the anterior, posterior and body groups, respectively. The common symptoms included dyskinesia (45, 42.5%), hypoesthesia (25, 23.6%), aphasia (21, 19.8%) and cognitive deficit (37, 34.9%). KPS scores were assessed finding 70 (66.0%) patients had scores over 70 scores, whereas 36 (34.0%) patients had scores under 70 scores. The NIHSS scores, ranging from 0 to 7 (mean, 2.19), were assessed preoperatively, confirming that 36 (34.0%) cases had a score of 0, 52 (49.1%) cases belonged to the 1–4 group and 18 (17.0%) cases belonged to the > 4 group. Pathological results showed that 31 (29.2%) and 75 (70.8%) cases were astrocytoma, WHO grade 4, IDH mutation and glioblastoma, respectively. The detailed clinical data are summarized in Table 1.

**Table 1:** The relationship between preoperative clinical data and EOR

Factors		Total	%	SMR	%	TR	%	***P*** value
**Total**		106		41	38.7%	65	61.3%	
**Gender**	Male	59	55.7%	25	42.4%	34	57.6%	0.382
	Female	47	44.3%	16	34.0%	31	66.0%	
**Age group**	18–47	40	37.7%	20	50.0%	20	50.0%	0.134
	48–63	51	48.1%	15	29.4%	36	70.6%	
	63–75	15	14.2%	6	40.0%	9	60.0%	
**Tumor location**	Anterior (Rostrum+Genu)	57	53.8%	24	42.1%	33	57.9%	0.544
	Posterior (Isthmus+Spleniu)	24	22.6%	7	29.2%	17	70.8%	
	Body	25	23.6%	10	40.0%	15	60.0%	
**Tumor volume (cm**^**3**^**)**	Mean + SD	55.2 ± 16.6		54.0 ± 16.9		56.0 ± 16.5		0.566
**Pathology**	Astrocytoma, WHO 4, IDH mutation	31	29.2%	14	45.2%	17	54.8%	0.378
	Glioblastoma	75	70.8%	27	36.0%	48	64.0%	
**KPS**	≥70	70	66.0%	27	38.6%	43	61.4%	0.975
	< 70	36	34.0%	14	38.9%	22	61.1%	
**NIHSS**	0	36	34.0%	18	50.0%	18	50.0%	0.190
	1–4	52	49.1%	16	30.8%	36	69.2%	
	> 4	18	17.0%	7	38.9%	11	61.1%	
**Dyskinesia**	Positive	45	42.5%	12	26.7%	33	73.3%	0.029
	Negative	61	57.5%	29	47.5%	32	52.5%	
**Hypoesthesia**	Positive	25	23.6%	6	24.0%	19	76.0%	0.085
	Negative	81	76.4%	35	43.2%	46	56.8%	
**Aphasia**	Positive	21	19.8%	8	38.1%	13	61.9%	0.951
	Negative	85	80.2%	33	38.8%	52	61.2%	
**Cognitive deficit**	Positive	37	34.9%	14	37.8%	23	62.2%	0.896
	Negative	69	65.1%	27	39.1%	42	60.9%	

*EOR* Extent of resection, *SMR* Supramaximal resection, *TR* Total resection, *SD* Standard deviation, *KPS* Karnofsky Performance Status scores, *NIHSS* National Institute of Health stroke scale.

### The en-bloc technique contributed to protecting postoperative neurologic function

The postoperative neurological deficits were represented by a decrease in KPS scores and NIHSS scores. A total of 6 patients (5.6%) had decreased KPS scores, and 24 patients (22.6%) had decreased NIHSS scores. Furthermore, we analyzed the effect of applying the en-bloc technique on the postoperative neurological complications by KPS scales and NIHSS scores. There was no significant difference in the change in KPS scores and NIHSS scores between the en-bloc subgroup and the no-en-bloc subgroup (*P* = 0.903 and 0.042, respectively, Table 2). However, due to the retrospective nature of this study, PSM analysis was applied to analyze the change in neurological function between the two groups to control for the mixed factors. The results showed that 5 patients (6.1%) had decreased KPS scores, whereas 21 patients (25.6%) had decreased NIHSS scores. Interestingly, as shown in Table 2, there was no significant difference in the change in KPS scores between the en bloc subgroup and the no-en bloc subgroup (*P* = 0.851), whereas there was a significant difference in the change in NIHSS scores between the two subgroups (*P* = 0.004). These results suggested that applying the en bloc technique did not result in an increase in new complications but rather had a positive effect on preserving or improving postoperative neurologic function.

**Table 2:** The effects of en-bloc resection on the postoperative complications

Factors	Unadjusted					Adjusted by PSM				
	En-bloc	%	No-En-bloc	%	***P*** value	En-bloc	%	No-En-bloc	%	***P*** value
**KPS**										
Improvement	9	36.0%	16	64.0%	0.903	9	47.4%	10	52.6%	0.851
No change	30	40.0%	45	60.0%		30	51.7%	28	48.3%	
Deterioration	2	33.3%	4	66.7%		2	40.0%	3	60.0%	
**NIHSS**										
Improvement	18	45.0%	22	55.0%	0.042	18	62.1%	11	37.9%	0.004
No change	19	45.2%	23	54.8%		19	58.1%	13	41.9%	
Deterioration	4	16.7%	20	83.3%		4	19.9%	17	80.1%	

*PSM* Propensity score matched analysis, *KPS* Karnofsky Performance Status scores.

* The differences were considered statistically significant for *P* < 0.025.

### The en-bloc technique raised the feasibility of supramaximal resection

As we described above, SMR was identified when FLAIR abnormalities were all removed in the relative lobe and all CE abnormalities were resected in the corpus callosum. EOR was subsequently analyzed according to this criterion. As shown in Table 3, SMR was achieved in 41 cases (38.7%), whereas TR was achieved in 65 cases (61.3%). Specifically, SMR was achieved in 28 cases (68.3%) and TR was achieved in 13 cases (31.7%) in the en-bloc series, whereas SMR was achieved in 13 cases (20.0%) and TR was achieved in 52 cases (80.0%) in the no-en-bloc series. Furthermore, a significant difference in EOR was observed between the en-bloc and the no-en-bloc subgroups, and a positive correlation was identified between EOR and the application of the en-bloc technique (*P* < 0.001, *r* = 0.483).

**Table 3:** En-bloc technique raised the feasibility of supramaximal resection

	Unadjusted							Adjusted by PSM						
	Total		En-bloc		No-En-bloc		***P*** value Correlation	Total		En-bloc		No-En-bloc		***P*** value Correlation
	***n***	%	***n***	%	***n***	%		***n***	%	***n***	%	***n***	%	
SMR	41	38.7%	28	68.3%	13	20.0%	*P* < 0.001 *r* = 0.483	28	34.1%	28	68.3%	0	0.0%	*P* < 0.001 *r* = 0.720
TR	65	61.3%	13	31.7%	52	80.0%		54	65.9%	13	31.7%	41	100.0%	

*PSM* Propensity score matched analysis, *SMR* Supramaximal resection, *TR* Total resection, *EOR* Extent of resection.

To control the confounders, PSM analysis was applied with a logistic regression, which was used with surgical technique as the dependent variable as a function of gender, age (continuous), tumor volume, tumor location, Pathology, preoperative KPS and preoperative NIHSS (continuous), and with 0.2 caliper width. A total of 82 cases were matched by PSM analysis. The results showed that SMR was achieved in 28 cases (34.1%), whereas TR was achieved in 54 cases (65.9%). In the en-bloc subgroup, SMR was achieved in 28 cases (68.3%) and TR was achieved in 13 cases (31.7%). In the no-en-bloc series, 0 cases and 41 cases (100.0%) achieved SMR and TR, respectively. More importantly, a statistical difference in EOR was also identified between the en-bloc subgroup and the no-en-bloc subgroup, and a positive correlation was identified between EOR and the application of the en-bloc technique (*P* < 0.001, *r* = 0.720). These unadjusted and adjusted results suggested that the en-bloc technique could contribute to supramaximal resection for ccGBM surgery.

### The en-bloc technique and supramaximal resection dramatically increased patient’s progression-free and overall survival

**Fig. 2:**
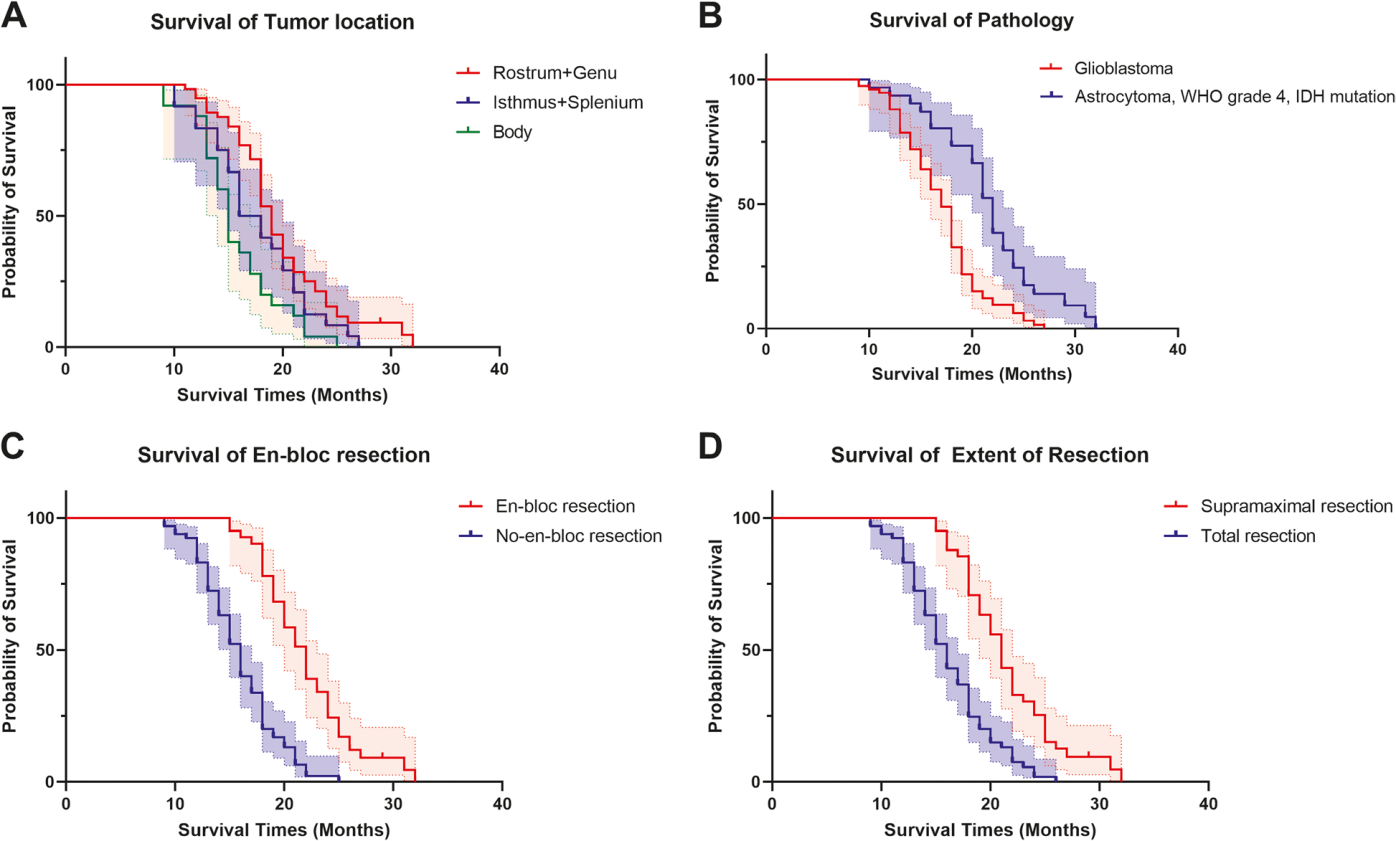
Overall survival curves of different tumor locations (**A**), pathologies (**B**), surgical strategies (**C**) and extents of resection (**D**)

**Fig. 3:**
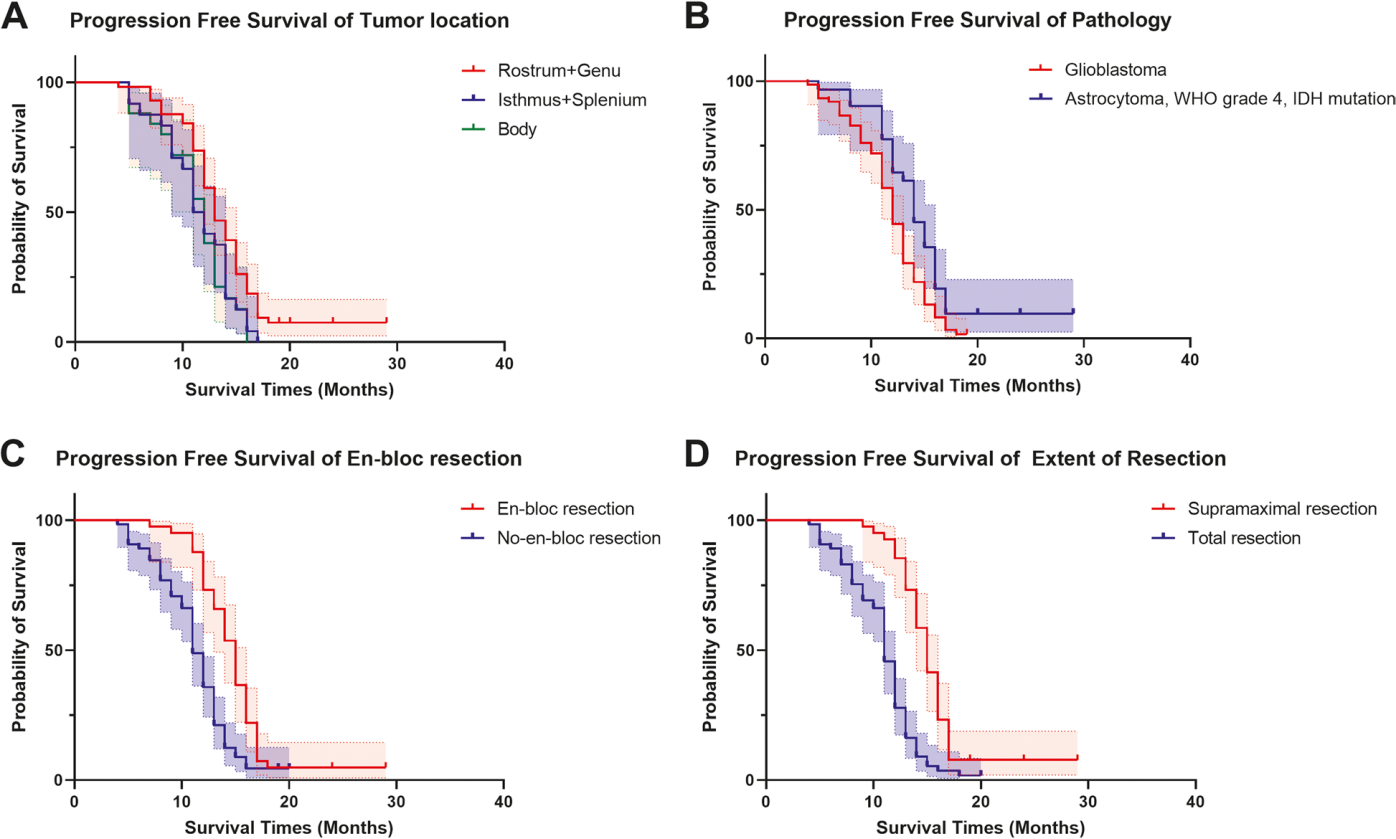
Progression-free survival curves of different tumor locations (**A**), pathologies (**B**), surgical strategies (**C**) and extents of resection (**D**)

The mean overall survival time and progression-free time were 18.305 ± 0.484 months (95% CI: 17.356–19.254 months) and 12.839 ± 0.460 months (95% CI: 11.934–13.737 months), respectively (Supplementary Table 2). Statistical results showed significant differences in OS and PFS among different tumor locations, different pathologies, application of the en-bloc technique and EOR (both *P* < 0.001) (Figs. 2 and 3). For different tumor locations, the mean survival and progression-free time of the anterior (rostrum+genu) subgroup were 19.761 ± 0.681 months (95% CI: 18.427–21.095 months) and 14.028 ± 0.697 months (95% CI: 12.661–15.395 months), the mean survival and progression-free time of the posterior (isthmus+spleniu) subgroup were 17.625 ± 0.973 months (95% CI: 15.718–19.532 months) and 11.500 ± 0.694 months (95% CI: 10.139–12.861 months), and the mean survival and progression-free time of the body subgroup were 15.737 ± 0.783 months (95% CI: 14.203–17.271 months) and 11.280 ± 0.671 months (95% CI: 9.964–12.596 months). For pathology, the mean survival and progression-free time of the astrocytoma, WHO grade 4, IDH mutation subgroup were 21.355 ± 1.025 months (95% CI: 19.346–23.365 months) and 14.806 ± 0.974 months (95% CI: 12.898–16.715 months), respectively, whereas the mean survival and progression-free time of the glioblastoma subgroup were 17.033 ± 0.467 months (95% CI: 16.118–17.947 months) and 11.817 ± 0.393 months (95% CI: 11.046–12.587 months), respectively. For en-bloc application, the mean survival and progression-free time of the en-bloc subgroup were 21.990 ± 0.675 months (95% CI: 2.667–23.313 months) and 14.854 ± 0.621 months (95% CI: 13.646–16.072 months), respectively, and the mean survival and progression-free time of the no-en-bloc subgroup were 15.888 ± 0.444 months (95% CI: 15.018–16.758 months) and 11.215 ± 0.442 months (95% CI: 10.349–12.081 months), respectively. The mean survival and progression-free time of the SMR subgroup were 21.625 ± 0.706 months (95% CI: 20.242–23.009 months) and 15.605 ± 0.689 months (95% CI: 14.254–16.956 months), respectively, whereas the mean survival and progression-free time of the TR subgroup were 16.194 ± 0.496 months (95% CI: 15.222–17.166 months) and 10.874 ± 0.406 months (95% CI: 10.078–11.670 months), respectively. These results suggested that patients with ccGBMs have a longer survival time and progression-free survival if the tumor is located in the anterior or posterior of the corpus callosum and have an IDH1 mutation, the en-bloc technique was applied, or supramaximal resection was achieved. Because of astrocytoma, WHO grade 4, IDH mutation and glioblastoma are different tumors according to the newest classification of gliomas, we additionally performed survival analysis in these two kinds of tumors, respectively. The results also confirmed that application of the en-bloc technique (Supplementary Fig. 1) significantly contributed to patients’ PFS and OS, indicating that patients with astrocytoma, WHO grade 4, IDH mutation or glioblastomas could benefit from the application of en-bloc technique.

Univariate analysis was applied to investigate the prognostic value of clinical characteristics (including gender, age groups, tumor volume, tumor location, different surgeons and pathology), EOR, en-bloc technique and preoperative and postoperative KPS and NIHSS scores on OS and PFS. As shown in Supplementary Table 3, tumor location, pathology, EOR, different surgeons and en-bloc technique were significantly associated with OS and PFS (both *P* < 0.05). Further multivariate analysis (as shown in Table 4) demonstrated that tumor location, pathology, EOR and en-bloc technique were significantly correlated with patient’s OS (both *P* < 0.05), and pathology, EOR and en-bloc technique were statistically correlated with patient’s PFS (both *P* < 0.05). However, different surgeons were not statistically correlated with patient’s OS and PFS in multivariate analysis. These results suggested that tumor location, pathology, EOR and en-bloc technique could be considered independent prognostic indicators of OS in patients with ccGBMs, and pathology, EOR and en-bloc technique could be considered independent prognostic indicators of PFS in patients with ccGBMs.

**Table 4:** Multivariate Cox regression analysis of overall survival and progression free survival

Factors	Overall survival			Progression free survival		
	***P***	HR	95% CI	***P***	HR	95% CI
**Location**						
Anterior (Rostrum+Genu)	< 0.001	0.370	0.221–0.618	0.032	/	/
Posterior (Isthmus+Spleniu)	0.008	0.447	0.247–0.810	0.195	/	/
Body	Reference			Reference		
**Pathology**						
Astrocytoma, WHO 4, IDH mutation	0.004	0.483	0.295–0.791	0.033	0.613	0.390–0.962
Glioblastoma	Reference			Reference		
**En-bloc technique**						
Yes	< 0.001	0.316	0.189–0.530	0.040	0.624	0.398–0.978
No	Reference			Reference		
**EOR**						
SMR	< 0.001	0.310	0.192–0.500	< 0.001	0.376	0.238–0.593
TR	Reference			Reference		

*HR* Hazard ratio, *CI* Confidence interval, *EOR* Extent of resection, *SMR* Supramaximal resection, *TR* Total resection, *KPS* Karnofsky Performance Status scores.

### The en-bloc technique significantly reduced the risk of recurrence in corpus callosum glioblastomas

To further confirm the essential role of the en-bloc technique on ccGBMs, we analyzed the interaction between the en-bloc technique and EOR. As shown in Table 5, the risk of recurrence of ccGBMs in which en-bloc resection was applied was significantly lower than that of ccGBMs in which en-bloc resection was not applied (HR: 0.444; 95% CI: 0.291–0.678; *P* < 0.001), and TR significantly increased the risk of recurrence compared to SMR (HR: 3.070; 95% CI: 1.985–4.747; *P* < 0.001). Notably, the en-bloc technique provided a marked risk reduction compared with the no-en-bloc technique (HR: 0.712; 95% CI: 0.535–0.947; *P* = 0.02) in patients undergoing TR, which was confirmed by our finding that there was a significant interactive effect between the en-bloc technique and EOR on progression-free survival.

**Table 5:** Effect of en-bloc technique on progression free survival in corpus callosum glioblastomas

Factors	Model 1		Model 2		Model 3		Model 4	
	Adjusted HR^a^ (95% CI)	***P***	Adjusted HR^a^ (95% CI)	***P***	Adjusted HR^a^ (95% CI)	***P***	Adjusted HR^a^ (95% CI)	***P***
**En-bloc technique**								
No	Reference				Reference			
Yes	0.444 (0.291–0.678)	< 0.001			0.624 (0.398–0.978)	0.040		
**EOR**								
SMR			Reference		Reference			
TR			3.070 (1.985–4.747)	< 0.001	2.660 (1.687–4.196)	< 0.001		
**Interaction effect**								
No-en-bloc technique + TR							Reference	
En-bloc technique + TR							0.712 (0.535–0.947)	0.020

^a^Cox proportional hazards model was adjusted for EOR, hydrocephalus, dissemination, preoperative KPS, preoperative dyskinesia, preoperative verbal deficit, preoperative cognitive deficit and postoperative seizure.

## Discussion

Corpus callosum glioblastoma is a specific type of GBM because of its essential anatomical location [18] and diverse molecular alterations [19]. Considering that the corpus callosum is the largest interhemispheric commissural tract in the deep brain and that tumors frequently invade the consecutive lobes, the risk of damage to critical anatomical structures and the incidence of severe neurological deficits are high, leading to poor postoperative quality of life. For this reason, the traditional strategy of ccGBMs is not recommended for radical resection [20]. Quite a number of patients underwent biopsy and subsequently received adjunctive chemoradiotherapy [1, 2, 4, 5], and the most prolonged median OS in those patients was only 7.2 months [6]. Therefore, ccGBMs have a poorer prognosis than GBMs without corpus callosum involvement.

It has been reported that 30% and 5 cm^3^ are defined as the maximum residual tumor proportion and volume, respectively, which are significantly associated with the prolonged survival and recurrence in high-grade gliomas [21]. However, a large number of studies have demonstrated the essential role of surgical resection in gliomas, and an enlarged EOR has positive effects on glioma patients’ survival, regardless of pathological type [22–25]. Based on this, we consider that the largest possible EOR for ccGBMs should be achieved. Corpus callosum glioblastoma can be divided into the corpus callosum and the lobar parts. To achieve a larger EOR in ccGBMs, we applied FLAIR-based resection in the lobar part, which recently has been confirmed to provide an extra longer survival for glioma patients [13, 26]. FLAIR-based resection means complete resection of abnormal signals in FLAIR images and can reduce tumor burden, resulting in a lower recurrence rate with no additional risk [11, 12, 27]. We found that supramaximal resection based on FLAIR images had a significant effect on patients’ PFS and OS.

En-bloc resection usually requires the presence of a tumoral envelope around the tumor, which is applied in several intracranial tumors, such as meningiomas, craniopharyngiomas [28], and pineal tumors [29] in our institution. However, the lack of a definite margin in gliomas results in the difficult application of the en-bloc technique [30]. However, as we previously described [14, 31], membranous structures, including the pia mater and ependyma, could be used as surgical margins because of their blocking effect on tumor cells. In addition, we consider that the en-bloc technique has several other advantages to glioma surgery. First, peritumoral resection contributes to protecting the critical arteries, veins, and nerves. Second, resection along the membranous structure margin can help to avoid dissemination caused by tumor fragmentation and reduce intraoperative bleeding. Third, the en-bloc technique can enhance the precision of intraoperative surgical guidance by avoiding shifts. Thus, the en-bloc technique has been applied in our center for many years. As a result, we found that the en-bloc technique contributed to an enlarged EOR and could subsequently increase PFS and OS in patients with ccGBMs. Interestingly, we also confirmed that the en-bloc technique reduced the risk of recurrence in patients with ccGBMs and provided a marked reduction in the risk of tumor recurrence compared with the no-en-bloc technique in tumors undergoing TR.

Maximal resection of ccGBMs has a higher risk of postoperative deficits. Previous studies reported that the risk rates of new complications in ccGBM surgery ranged from 17.2 to 42.9% [1, 2, 4–6], and a recent meta-analysis study revealed that the odds ratio of neurologic complications was 2.05 in patients undergoing surgical resection compared to those undergoing biopsy [32]. Moreover, because the selection of patients with ccGBMs for surgical resection had inherent bias and the present conservative strategy of ccGBM surgery, the real incidence of postoperative neurologic deficits should have a higher rate [3]. However, we believe that the high risk of postoperative complications is determined by the characteristics of ccGBMs rather than surgical resection. Our study confirmed that surgical resection of ccGBMs had an acceptable risk of postoperative neurologic deficits. Applying the en-bloc technique did not increase the incidence of new complications and had a positive effect on preserving or improving postoperative neurologic function. Based on this, we considered that maximal resection could be achieved safely in patients with ccGBMs.

This study confirmed the essential roles of en-bloc-based maximal resection in patients with ccGBMs. However, our work had several limitations. First, it was a retrospective study in a single center rather than a randomized clinical trial in multiple centers. Because of the features of ccGBMs, an internal bias in the selection of applying the en-bloc technique was unavoidable. For instance, we preferred to apply resection based on T2-FLAIR in tumors located the anterior and the posterior of the corpus callosum because tumors located in the body of the corpus callosum usually involve the critical functional area. Second, we had three different surgeons perform two surgical methods, which could result in an external bias for this study. Third, our series had limited cases, indicating that further investigation should be considered based on a more extensive cohort. Finally, the insufficient molecular status profiles are lacking, which would have important value in clinical practice.

## Conclusions

In conclusion, we demonstrated that applying the en-bloc technique had a positive effect on preserving or improving postoperative neurologic function compared to patients with ccGBMs without en-bloc resection. A positive correlation between EOR and the en-bloc technique indicated that applying the en-bloc technique could contribute to the enlarged maximal resection. Further survival analysis confirmed that applying the en-bloc technique and achieving supramaximal resection could significantly prolong OS and PFS, and multivariate analysis suggested that tumor location, pathology, EOR and the en-bloc technique could be regarded as independent prognostic indicators for OS in patients with ccGBMs, and pathology, EOR and the en-bloc technique were independently correlated with patient’s PFS. Remarkably, the en-bloc technique also provided a marked reduction in the risk of tumor recurrence compared with the no-en-bloc technique in tumors undergoing TR, indicating the essential role of the en-bloc technique in ccGBM surgery.

### Supplementary Information

**Additional file 1: Supplementary Tables.**

**Additional file 2: Supplementary Figure 1.**

## Data Availability

The datasets analyzed during the current study are not publicly available due to patients’ privacy but are available from the corresponding author on reasonable request.

## Abbreviations

ccGBM: Corpus callosum glioblastoma
EOR: Extent of resection
SMR: Supramaximal resection
TR: Total resection
STR: Subtotal resection
MRI: Magnetic resonance imaging
CE: Contrast-enhanced
DWI: Diffusion-weighted imaging
FLAIR: Fluid Attenuated Inversion Recovery
OS: Overall survival
PFS: Progression-free survival
CI: Confidence interval
HR: Hazard ratio
PSM: Propensity score matching
NIHSS: National Institute of Health stroke scale
KPS: Karnofsky Performance Status
