# Supplementary material for: Applying the en-bloc technique in corpus callosum glioblastoma surgery contributes to maximal resection and better prognosis: a retrospective study
Source: BMC Surg. 2024 Jan 2;24:4. doi: 10.1186/s12893-023-02264-4 (PMC10763443; doi:10.1186/s12893-023-02264-4)
Supplement: Supplementary file 1 — Additional file 1: Supplementary Tables. [file 12893_2023_2264_MOESM1_ESM.docx]

Supplementary Tables

**Supplementary Table 1.** **The relationship between preoperative clinical data and technique of resection**

| **Factors** | | **Total** | **%** | **En-bloc** | **%** | **No-En-bloc** | **%** | ***P* value** |
| --- | --- | --- | --- | --- | --- | --- | --- | --- |
| **Total** | | 106 |  | 41 | 38.7% | 65 | 61.3% |  |
| **Gender** | Male | 59 | 55.7% | 24 | 40.7% | 35 | 59.3% | 0.636 |
|  | Female | 47 | 44.3% | 17 | 36.2% | 30 | 63.8% |  |
| **Age group** | 18-47 | 40 | 37.7% | 17 | 42.5% | 23 | 57.5% | 0.531 |
|  | 48-63 | 51 | 48.1% | 17 | 33.3% | 34 | 66.7% |  |
|  | 63-75 | 15 | 14.2% | 7 | 46.7% | 8 | 53.3% |  |
| **Tumor location** | Anterior (Rostrum+Genu) | 57 | 53.8% | 27 | 47.4% | 30 | 52.6% | 0.140 |
|  | Posterior (Isthmus+Spleniu) | 24 | 22.6% | 7 | 29.2% | 17 | 70.8% |  |
|  | Body | 25 | 23.6% | 7 | 28.0% | 18 | 72.0% |  |
| **Tumor volume (cm^3^)** | Mean + SD | 55.2 ± 16.6 |  | 51.5 ± 14.0 |  | 57.6 ± 17.7 |  | 0.066 |
| **Pathology** | Astrocytoma, WHO 4, IDH mutation | 31 | 29.2% | 18 | 58.1% | 13 | 41.9% | 0.008 |
|  | Glioblastoma | 75 | 70.8% | 23 | 30.7% | 52 | 69.3% |  |
| **KPS** | ≥70 | 70 | 66.0% | 30 | 42.9% | 40 | 57.1% | 0.218 |
|  | <70 | 36 | 34.0% | 11 | 30.6% | 25 | 69.4% |  |
| **NIHSS** | 0 | 36 | 34.0% | 18 | 50.0% | 18 | 50.0% | 0.229 |
|  | 1-4 | 52 | 49.0% | 17 | 32.7% | 35 | 67.3% |  |
|  | >4 | 18 | 17.0% | 6 | 33.3% | 12 | 66.7% |  |
| **Dyskinesia** | Positive | 45 | 42.5% | 12 | 26.7% | 33 | 73.3% | 0.029 |
|  | Negative | 61 | 57.5% | 29 | 47.5% | 32 | 52.5% |  |
| **Hypoesthesia** | Positive | 25 | 23.6% | 7 | 28.0% | 18 | 72.0% | 0.210 |
|  | Negative | 81 | 76.4% | 34 | 42.0% | 47 | 58.0% |  |
| **Aphasia** | Positive | 21 | 19.8% | 7 | 33.3% | 14 | 66.7% | 0.574 |
|  | Negative | 85 | 80.2% | 34 | 40.0% | 51 | 60.0% |  |
| **Cognitive deficit** | Positive | 37 | 34.9% | 14 | 37.8% | 23 | 62.2% | 0.896 |
|  | Negative | 69 | 65.1% | 27 | 39.1% | 42 | 60.9% |  |

EOR: Extent of resection; SMR: Supramaximal resection; TR: Total resection; KPS: Karnofsky Performance Status scores; NIHSS: National Institute of Health stroke scale.

**Supplementary** **Table 2. Overall survival and** **progression free survival time**

| **Factors** | | **Overall survival** | | | |  | **Progression free survival** | | | |
| --- | --- | --- | --- | --- | --- | --- | --- | --- | --- | --- |
|  |  | **Mean (Months)** | **SD** | **95%CI** | |  | **Mean (Months)** | **SD** | **95%CI** | |
|  |  |  |  | **Lower** | **Upper** |  |  |  | **Lower** | **Upper** |
| **Total cases** | | 18.305 | 0.484 | 17.356 | 19.254 |  | 12.836 | 0.460 | 11.934 | 13.737 |
| **Location** | Anterior (Rostrum+Genu) | 19.761 | 0.681 | 18.427 | 21.095 |  | 14.028 | 0.697 | 12.661 | 15.395 |
|  | Posterior (Isthmus+Spleniu) | 17.625 | 0.973 | 15.718 | 19.532 |  | 11.500 | 0.694 | 10.139 | 12.861 |
|  | Body | 15.737 | 0.783 | 14.203 | 17.271 |  | 11.280 | 0.671 | 9.964 | 12.596 |
| **Pathology** | Astrocytoma, WHO 4, IDH mutation | 21.355 | 1.025 | 19.346 | 23.365 |  | 14.806 | 0.974 | 12.898 | 16.715 |
|  | Glioblastoma | 17.033 | 0.467 | 16.118 | 17.947 |  | 11.817 | 0.393 | 11.046 | 12.587 |
| **En-bloc technique** | Yes | 21.990 | 0.675 | 20.667 | 23.313 |  | 14.854 | 0.621 | 13.636 | 16.072 |
|  | No | 15.888 | 0.444 | 15.018 | 16.758 |  | 11.215 | 0.442 | 10.349 | 12.081 |
| **EOR** | SMR | 21.625 | 0.706 | 20.242 | 23.009 |  | 15.605 | 0.689 | 14.254 | 16.956 |
|  | TR | 16.194 | 0.496 | 15.222 | 17.166 |  | 10.874 | 0.406 | 10.078 | 11.670 |

EOR: Extent of resection; SMR: Supramaximal resection; TR: Total resection; SD: Standard deviation; CI: Confidence interval.

**Supplementary Table 3. Univariate Cox regression analysis of overall survival and progression free survival**

| **Factors** | **Overall survival** | | |  | **Progression free survival** | | |
| --- | --- | --- | --- | --- | --- | --- | --- |
|  | ***P*** | **HR** | **95% CI** |  | ***P*** | **HR** | **95% CI** |
| **Location** |  |  |  |  |  |  |  |
| Anterior (Rostrum+Genu) | **0.001** | 0.428 | 0.262-0.698 |  | **0.025** | 0.561 | 0.339-0.931 |
| Posterior (Isthmus+Spleniu) | 0.095 | 0.617 | 0.350-1.087 |  | 0.824 | 0.937 | 0.528-1.664 |
| Body | Reference |  |  |  | Reference |  |  |
| **Tumor volume** | 0.475 | 1.005 | 0.992-1.017 |  | 0.062 | 0.988 | 0.976-1.001 |
| **Pathology** |  |  |  |  |  |  |  |
| Astrocytoma, WHO 4, IDH mutation | **<0.001** | 0.398 | 0.250-0.634 |  | **0.016** | 0.577 | 0.370-0.901 |
| Glioblastoma | Reference |  |  |  | Reference |  |  |
| **En-bloc technique** |  |  |  |  |  |  |  |
| Yes | **<0.001** | 0.240 | 0.150-0.384 |  | **<0.001** | 0.444 | 0.291-0.678 |
| No | Reference |  |  |  | Reference |  |  |
| **Surgeons** |  |  |  |  |  |  |  |
| A | **0.001** | 2.409 | 1.453-3.995 |  | **0.026** | 1.742 | 1.070-2.838 |
| B | **0.006** | 2.076 | 1.233-3.496 |  | **0.003** | 2.183 | 1.293-3.685 |
| C | Reference |  |  |  | Reference |  |  |
| **EOR** |  |  |  |  |  |  |  |
| SMR | **<0.001** | 0.326 | 0.211-0.503 |  | **<0.001** | 0.330 | 0.214-0.510 |
| TR | Reference |  |  |  | Reference |  |  |
| **Pre-KPS** |  |  |  |  |  |  |  |
| ≥70 | 0.557 | 1.134 | 0.745-1.725 |  | 0.725 | 1.078 | 0.708-1.641 |
| <70 | Reference |  |  |  | Reference |  |  |
| **Post-KPS** |  |  |  |  |  |  |  |
| ≥70 | 0.094 | 1.584 | 0.924-2.716 |  | 0.187 | 1.436 | 0.839-2.458 |
| <70 | Reference |  |  |  | Reference |  |  |
| **Pre-NIHSS** |  |  |  |  |  |  |  |
| 0 | 0.091 | 0.584 | 0.313-1.090 |  | 0.686 | 1.133 | 0.618-2.076 |
| 1-4 | 0.640 | 0.873 | 0.493-1.545 |  | 0.245 | 1.399 | 0.794-2.466 |
| >4 | Reference |  |  |  | Reference |  |  |
| **Post-NIHSS** |  |  |  |  |  |  |  |
| 0 | **0.017** | 0.450 | 0.233-0.867 |  | **0.038** | 0.499 | 0.258-0.963 |
| 1-4 | 0.469 | 0.789 | 0.416-1.499 |  | 0.328 | 0.726 | 0.382-1.379 |
| >4 | Reference |  |  |  | Reference |  |  |

HR: Hazard ratio; CI: Confidence interval; EOR: Extent of resection; SMR: Supramaximal resection; TR: Total resection; KPS: Karnofsky Performance Status scores; NIHSS: National Institute of Health stroke scale.
