# Supplementary material for: Applying the en-bloc technique in corpus callosum glioblastoma surgery contributes to maximal resection and better prognosis: a retrospective study
Source: BMC Surg. 2024 Jan 2;24:4. doi: 10.1186/s12893-023-02264-4 (PMC10763443; doi:10.1186/s12893-023-02264-4)
Supplement: Supplementary file 2 — Additional file 2: Supplementary Figure 1. [file 12893_2023_2264_MOESM2_ESM.docx]

**
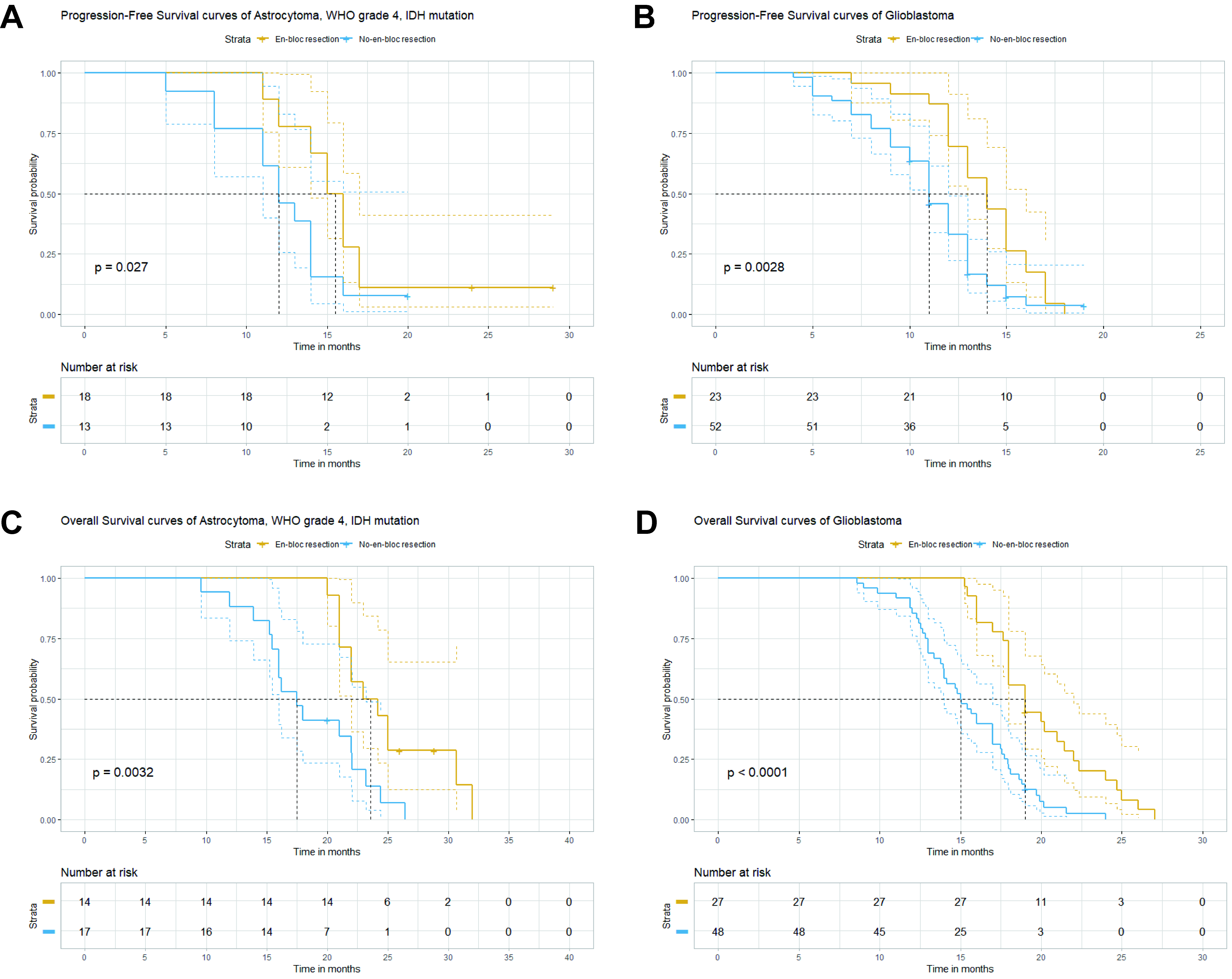
**

**Supplementary Figure 1.** Progression-free survival curves of different technique of resection in astrocytoma, WHO grade 4, IDH mutation (**A**) and glioblastoma (**B**); Overall survival curves of different technique of resection in astrocytoma, WHO grade 4, IDH mutation (**C**) and glioblastoma (**D**).
